# Supplementary material for: Hydrogen peroxide release by bacteria suppresses inflammasome-dependent innate immunity
Source: Nat Commun. 2019 Aug 2;10:3493. doi: 10.1038/s41467-019-11169-x (PMC6677825; doi:10.1038/s41467-019-11169-x)
Supplement: Supplementary file 1 — Supplementary Information [file 41467_2019_11169_MOESM1_ESM.pdf]

Supplementary Information:

Hydrogen peroxide release by bacteria suppresses inflammasome-dependent innate immunity

Erttmann S.F. and Gekara N.O.

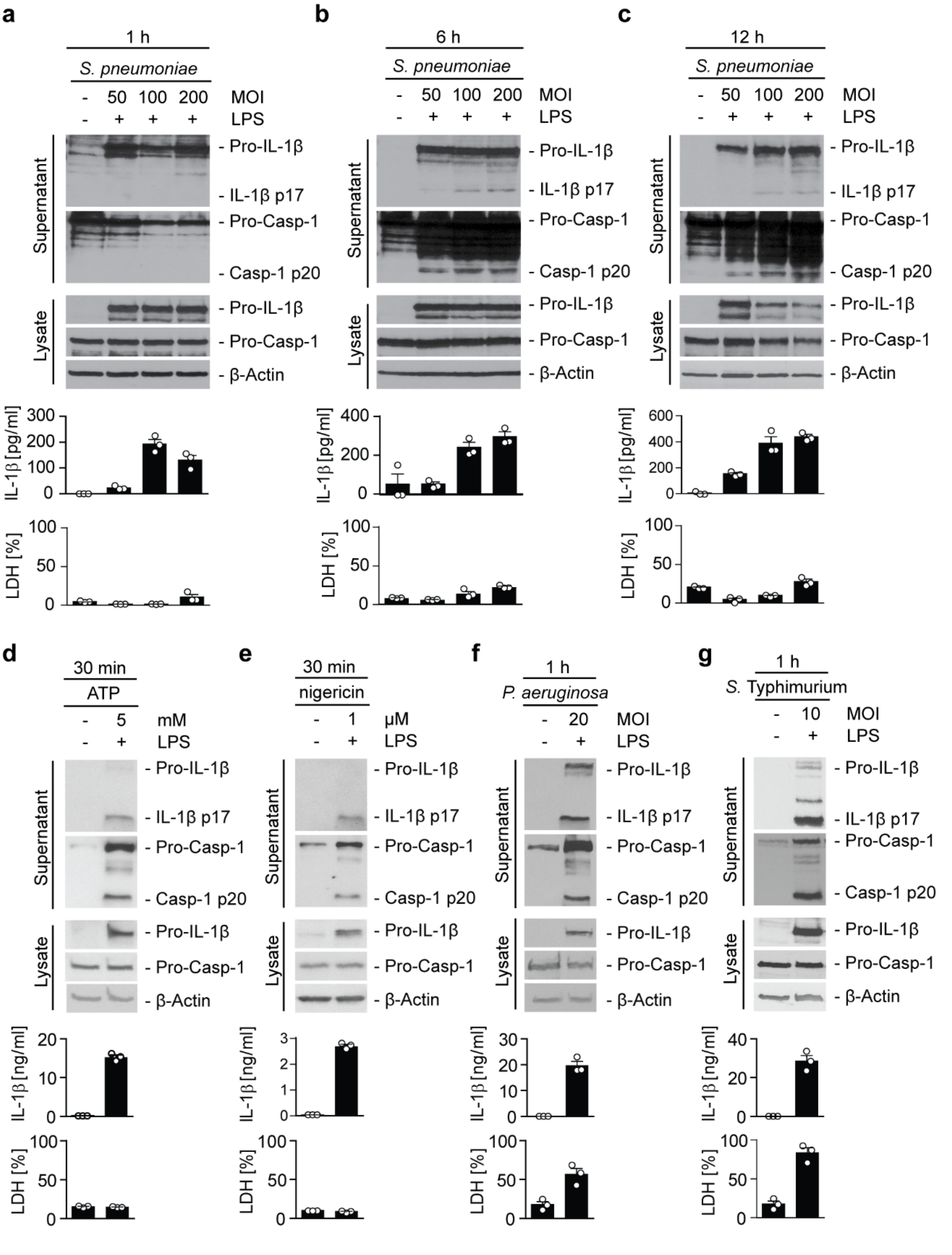

Supplementary Figure 1 *S. pneumoniae* infection is associated with weak and delayed inflammasome activation. a-c Caspase-1 and IL-1β processing, IL-1β release measured by ELISA, and LDH release in BMDMs primed (or not) with LPS for 4.5 h and subsequently infected with *S. pneumoniae* D39 (WT) at MOI 50, 100 and 200 for 1 h **a**, 6 h **b** or 12 h **c**. **d-g** Caspase-1 and IL-1β processing, IL-1β and LDH release in LPS-primed BMDMs stimulated with **d** ATP (5 mM) or **e** nigericin (1 μM) for 30 min or infected with **f** *P. aeruginosa* (MOI 20) or **g** *S.* Typhimurium (MOI 10) for 60 min. Immunoblot results are representative of 3 independent experiments. IL-1β and LDH release data are depicted as mean ± standard error of the mean (s.e.m.) of three independent experiments. Source data are provided as a Source Data file.

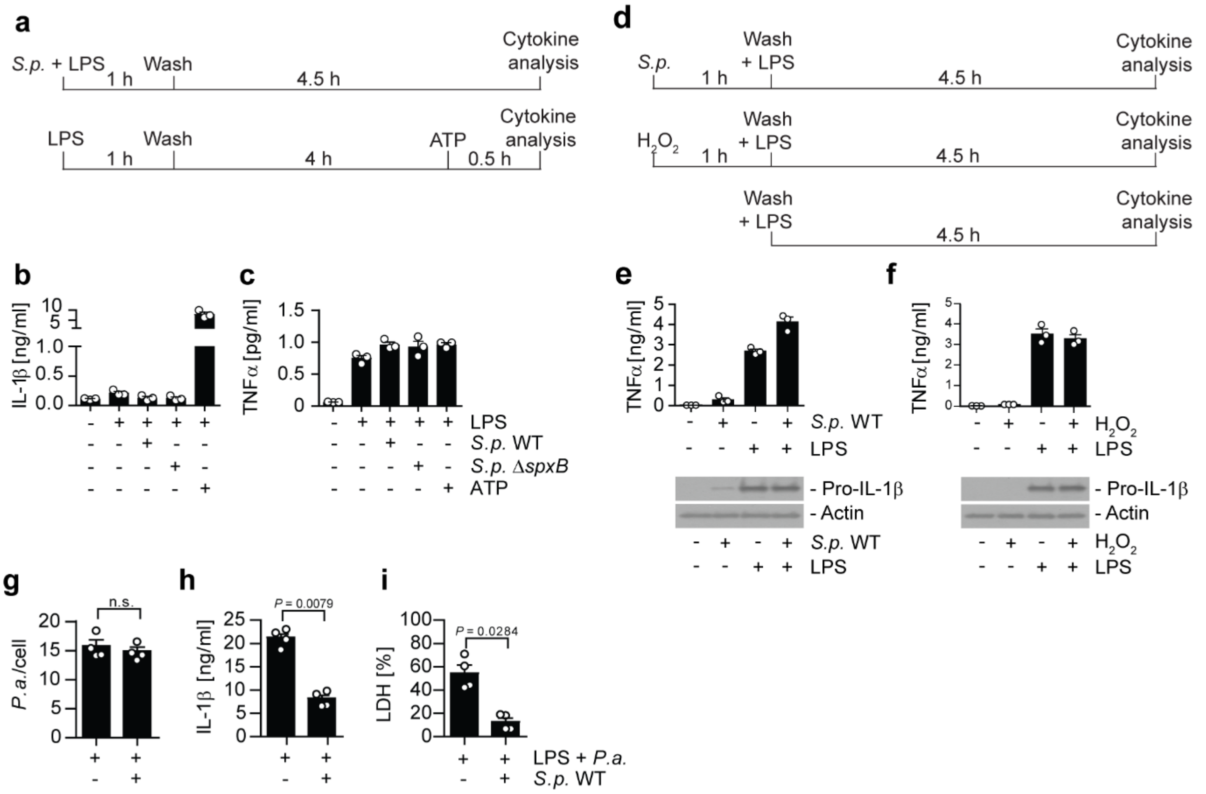

Supplementary Figure 2 Inflammasome inhibition by *S. pneumoniae* is not due to impaired priming, phagocytosis or loss of macrophage viability. a Schematic illustration of the experimental layout in **b** and **c**. **b**, **c** ELISA for IL-1β and TNFα on supernatants of BMDMs stimulated with LPS (500 ng mL^{-1}) or simultaneously stimulated with *S.p.* WT (D39, MOI 100) or *S.p.* Δ*spxB* (MOI 100) and LPS. LPS + ATP stimulation was included as control. ELISA data are presented as the mean ± s.e.m. of three independent experiments. **d** Schematic illustration of the experimental layout in **e** and **f**. **e**, **f** ELISA for TNFα on supernatants and immunoblot analysis for pro-IL-1β on cell lysates from BMDMs pre-treated with *S.p*. WT (MOI 100) **e** or 50 µM H_2O_2 **f** for 1 h followed by stimulation with LPS for 4.5 h. Immunoblots are representative of three independent experiments, ELISA data are presented as the mean ± s.e.m. of 3 independent experiments. **g** Number of intracellular *P. aeruginosa* per cell in LPS-primed BMDM pre-infected or not with *S. pneumoniae* (D39, MOI 100, 30 min) and subsequently infected with *P. aeruginosa* (MOI 20, 60 min). **h** Analysis of supernatants for IL-1β secretion and **i** LDH release. Results of four independent experiments; data are shown as mean ± s.e.m.. *P* values in **g** to **i** are determined by Mann Whitney test; n.s.: not significant. Source data are provided as a Source Data file.

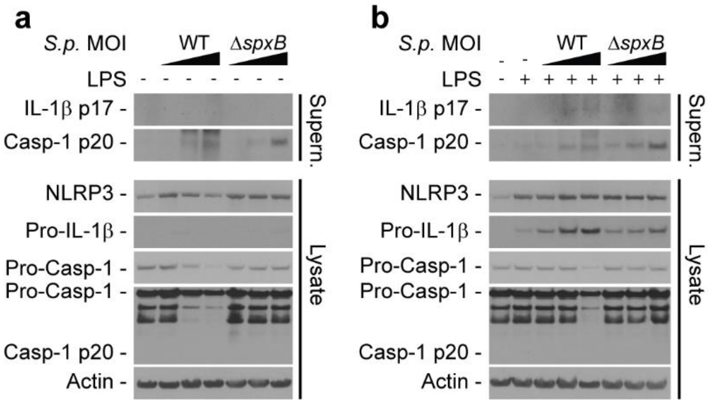

Supplementary Figure 3 Long-term infection with *S. pneumoniae* Δ*spxB* results in more robust inflammasome activation than infection with wild-type *S. pneumoniae*. a Immunoblot analysis of IL-1β and Caspase-1 processing in un-primed BMDMs infected with *S.p.* WT or Δ*spxB* (D39) at MOI 50, 100 or 200 for 12 h. **b** Immunoblot analysis of IL-1β and Caspase-1 processing in BMDMs primed with 500 ng/ml LPS for 4 h and then infected with *S.p.* WT or Δ*spxB* (D39) at MOI 50, 100 or 200 for 12 h. Immunoblots in **a** and **b** are representative of two independent experiments. Source data are provided as a Source Data file.

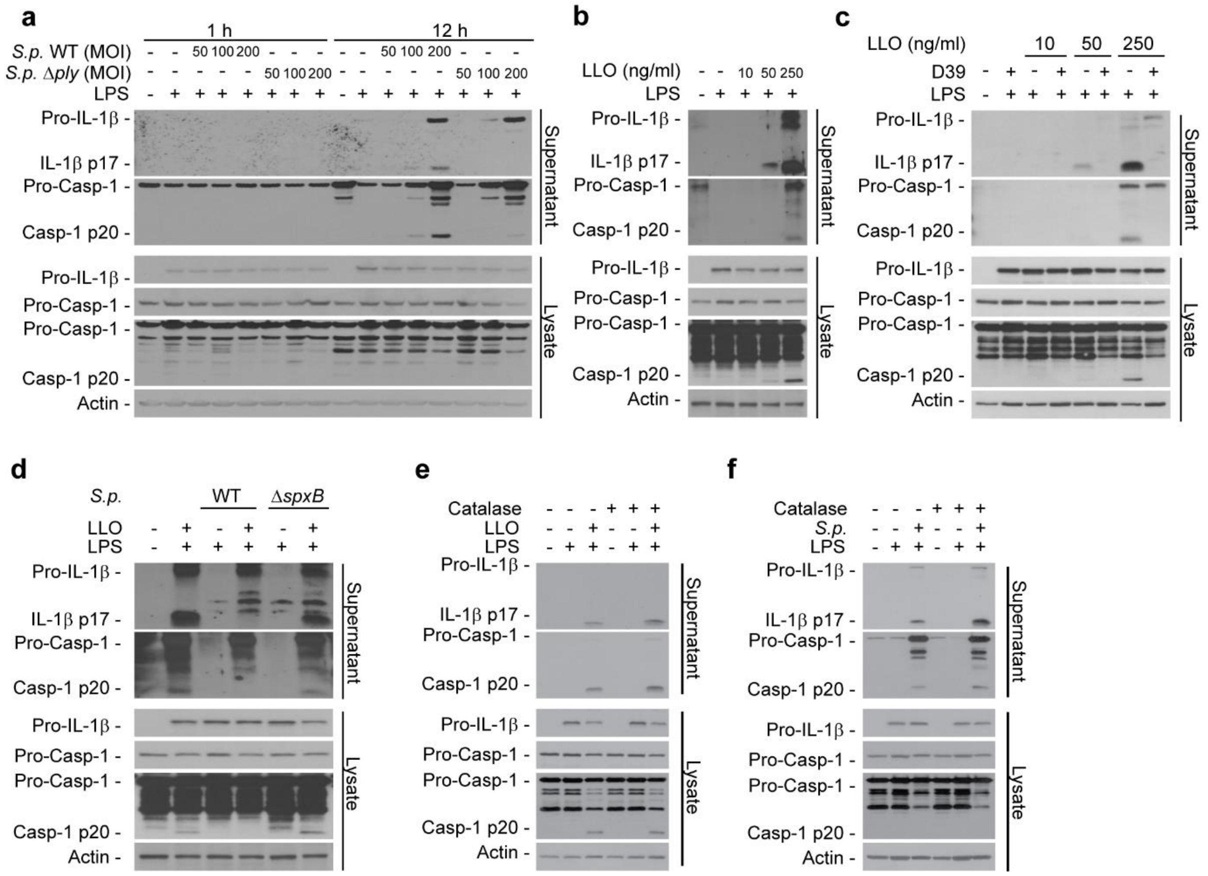

Supplementary Figure 4 ***S. pneumoniae*** **inhibits inflammasome activation induced by thiol-dependent pore forming toxin**. **a** PLY is essential for inflammasome activation by *S. pneumoniae*. Immunoblot analysis of IL-1β and Caspase-1 processing in LPS-primed BMDMs infected with *S.p.* WT or Δ*ply* (D39) at MOI 50, 100 or 200 for 1 or 12 h. Immunoblots are representative of 2 independent experiments. **b** Immunoblot analysis of IL-1β and Caspase-1 processing in LPS-primed BMDMs stimulated with 10, 50 or 250 ng mL^{-1} listeriolysin O (LLO) for 45 min. **c** Immunoblot analysis of IL-1β and Caspase-1 processing in LPS-primed BMDMs pre-infected with *S.p.* WT (D39, MOI 50, 30 min) and then stimulated with 10, 50 or 250 ng mL^{-1} LLO for 45 min. **d** Immunoblot analysis of IL-1β and Caspase-1 processing in LPS-primed BMDMs pre-infected with *S.p.* WT or *S.p.* Δ*spxB* (D39, MOI 50, 30 min) and then stimulated with 250 ng mL^{-1} LLO for 45 min. Immunoblots in **b** to **d** are representative of 3 or 4 independent experiments, respectively. **e** Immunoblot analysis of IL-1β and Caspase-1 processing in LPS-primed BMDMs stimulated with 250 ng mL^{-1} LLO for 60 min in the presence or absence of 100 U mL^{-1} catalase. **f** Immunoblot analysis of IL-1β and Caspase-1 processing in LPS-primed BMDMs infected with *S.p.* WT (D39, MOI 200) for 12 h in the presence or absence of 100 U mL^{-1} catalase during the last 3 h of infection. Immunoblots in **e** and **f** are representative of two independent experiments. Source data are provided as a Source Data file.

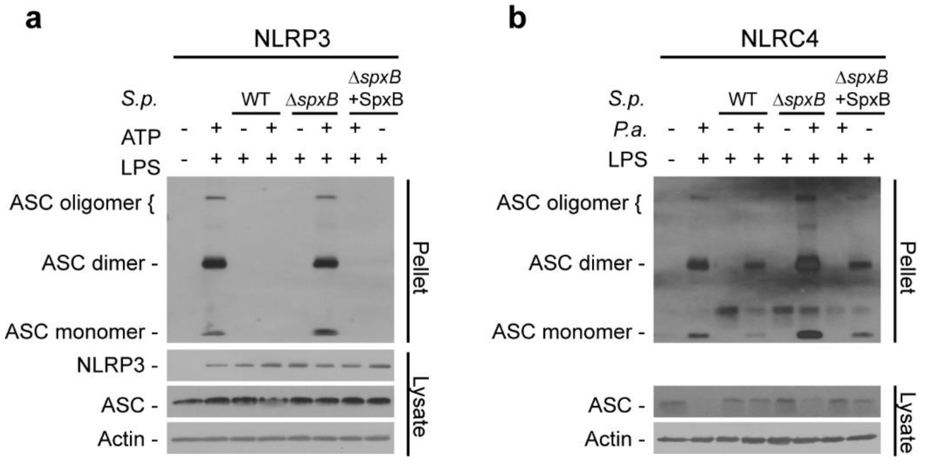

Supplementary Figure 5 Inflammasome inhibition by *S. pneumoniae* involves defects in ASC complex formation. a, b Immunoblot analysis of ASC oligomers in LPS-primed BMDMs pre-treated with *S.p.* WT (D39), *S.p.* Δ*spxB* or *S.p.* SpxB-complemented Δ*spxB* (Δ*spxB* + SpxB) (MOI 50) for 30 min followed by **a** stimulation with 5 mM ATP for 30 min or **b** infection with *P.a.* (MOI 20) for 60 min. The results are representative of two independent experiments. Source data are provided as a Source Data file.

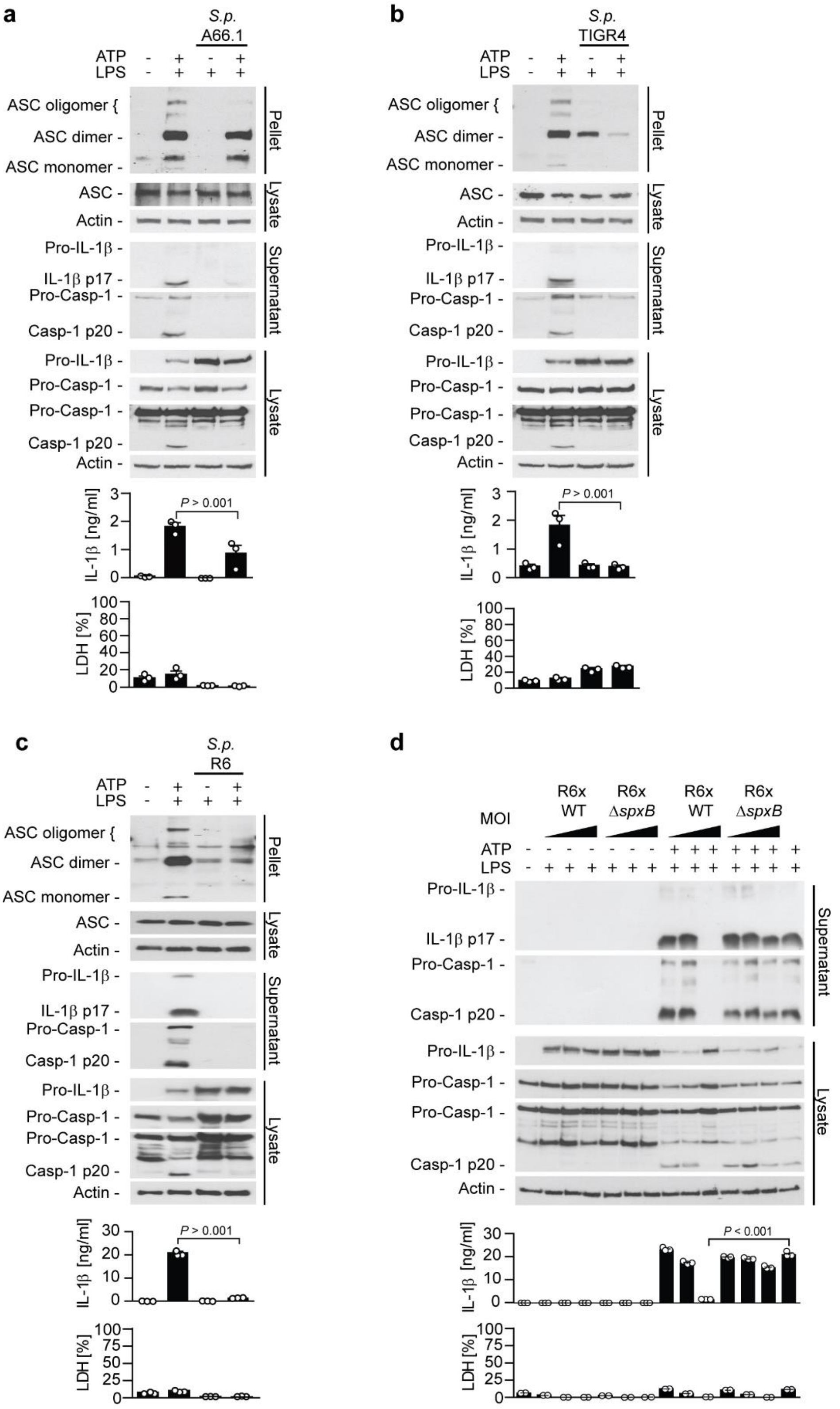

Supplementary Figure 6 Inhibition of inflammasome activation by *S. pneumoniae* is not strain-specific. **a-c** Immunoblot analysis of ASC oligomers, Caspase-1, and IL-1β processing in LPS-primed BMDMs pre-treated with **a** *S. pneumoniae* A66.1, **b** TIGR4 or **c** R6 (MOI 50) for 30 min before stimulation with ATP (5 mM) for 30 min. **d** Immunoblot analysis of Caspase-1 and IL-1β processing in LPS-primed BMDMs pre-treated with raising MOIs of *S. pneumoniae* R6x WT or R6x Δ*spxB* (MOI 0.5, 5, 50 for 30 min) before stimulation with ATP for 30 min. Representative results of three independent experiments. **a - d** Analyses of corresponding supernatants for IL-1β secretion by ELISA and LDH release; the data are shown as the mean ± s.e.m. of three independent experiments. *P* values are determined by one-way ANOVA followed by Bonferroni post-test. Source data are provided as a Source Data file.

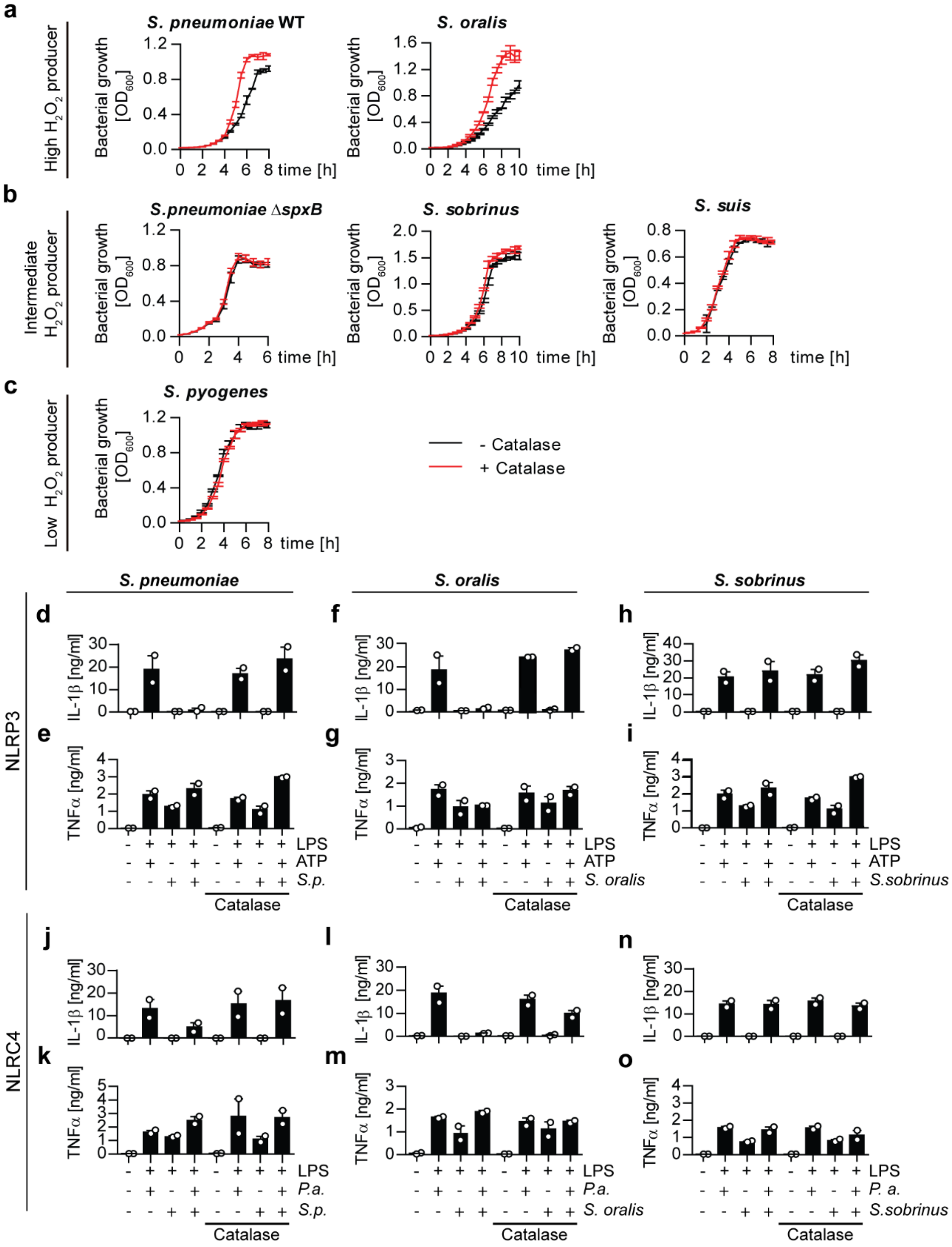

Supplementary Figure 7 Catalase abolishes inflammasome inhibition by high H_2O_2-producing bacteria. a-c *In vitro* growth rate of **a** *S. pneumoniae* WT (D39) and *S. oralis*, **b** *S. pneumoniae* Δ*spxB*, *S. sobrinus*, and *S. suis* and **c** *S. pyogenes* in the presence or absence of catalase (100 U mL^{-1}). Data are representative of three independent experiments each performed in triplicates; the data are shown as the mean ± s.d.. **d-o** ELISA analysis of supernatants for IL-1β and TNFα secretion of LPS-primed BMDMs pre-treated with **d**, **e**, **j**, **k** *S. pneumoniae* WT (D39), **f**, **g**, **l**, **m** *S. oralis* or **h**, **i**, **n**, **o** *S. sobrinus* at MOI 40 for 30 min in the presence or absence of catalase (100 U mL^{-1}) before stimulation with **d-i** ATP (5 mM) for 30 min or infection with **j-o** *P. aeruginosa* (MOI 20) for 60 min. Data represent two independent experiments depicted as the mean ± s.e.m.. Source data are provided as a Source Data file.

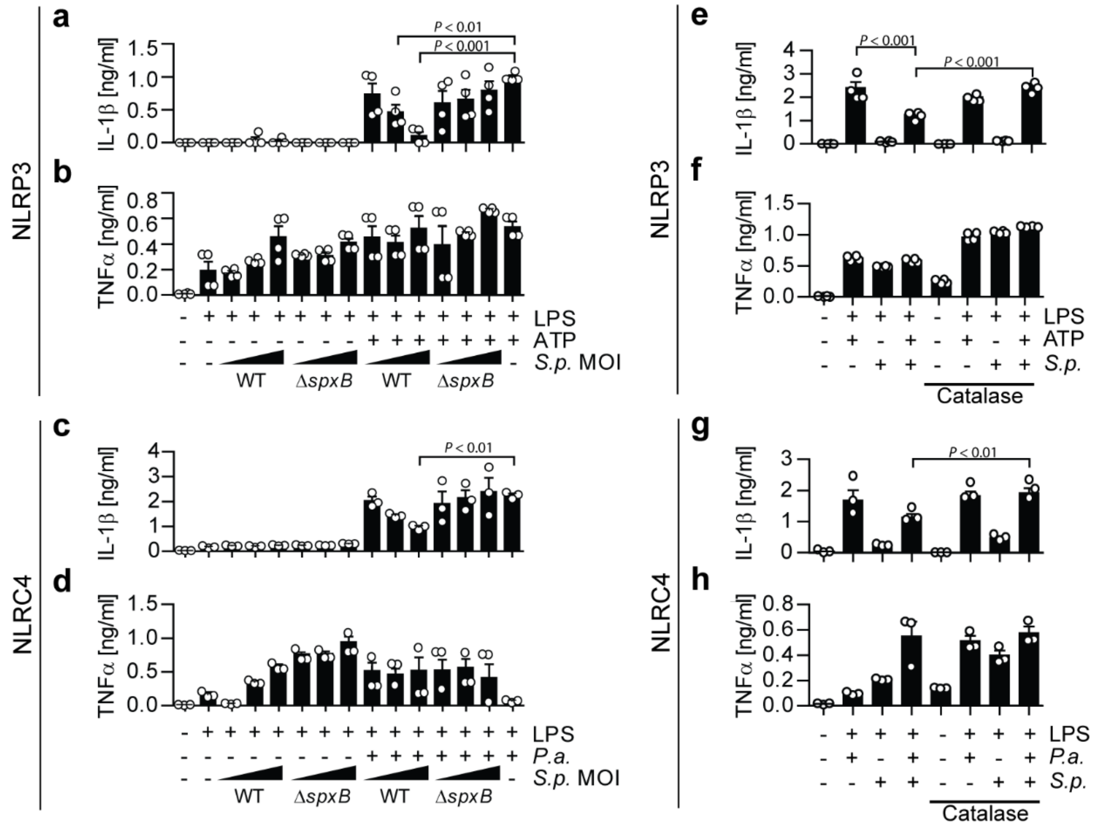

Supplementary Figure 8 H_2O_2 release by *S. pneumoniae* inhibits inflammasome activation in human PBMCs. a-d ELISA analysis of supernatants for **a**, **c** IL-1β and **b**, **d** TNF-α secreted by human PBMCs pre-treated with increasing doses (MOIs 0.5, 5 or 50) of *S.p.* WT or *S.p.* Δ*spxB* (D39) for 30 min before stimulation with **a**, **b** ATP for 30 min or infection with **c**, **d** *P. aeruginosa* (*P.a.*, MOI 20, 60 min). Depicted results are obtained from three or four independent experiments, respectively. **e-h** ELISA analysis of supernatants for **e**, **g** IL-1β and **f**, **h** TNF-α secreted by LPS-primed human PBMCs pre-infected with *S.p.* WT (D39, MOI 50) in the presence or absence of catalase (100 U mL^{-1}) for 30 min, before stimulation with **e**, **f** 5 mM ATP for 30 min or infection with **g**, **h** *P.a.* at MOI 20 for 60 min. Results of three or four independent experiments, respectively, are shown as the mean ± s.e.m.. *P* values in **a** to **h** are determined by one-way ANOVA followed by Bonferroni post-test.

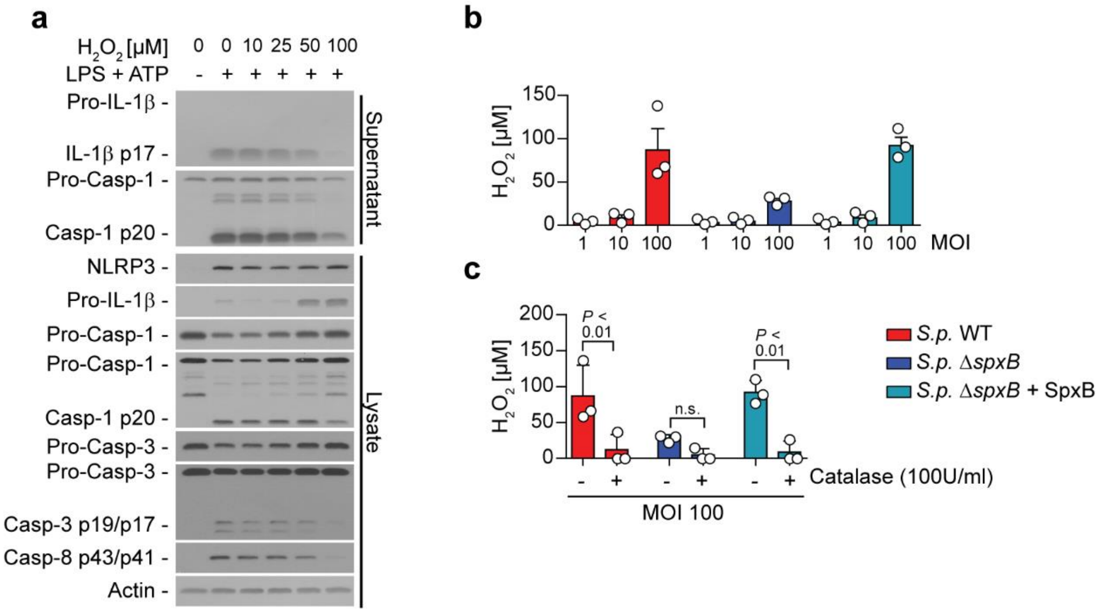

Supplementary Figure 9 H_2O_2 inhibits inflammasome activation in a dose-dependent manner. a Immunoblot analysis of Caspase 1, IL-1β, Caspase-3 and -8 processing in LPS-primed BMDMs pre-treated with 10 – 100 μM H_2O_2 for 10 min and then stimulated with 5 mM ATP for 30 min. The results are representative of two independent experiments. **b** H_2O_2 concentration released by *S.p.* WT (D39), *S.p.* Δ*spxB*, and *S.p.* Δ*spxB* + SpxB after BMDM infection at MOI 1, 10 and 100 for 1 h. **c** H_2O_2 concentration released by *S.p.* WT (D39), *S.p.* Δ*spxB*, and *S.p.* Δ*spxB* + SpxB after BMDM infection at MOI 100 for 1 h in the presence or absence of 100 U mL^{-1} catalase. Results in **b** and **c** represent the mean ± s.e.m. of three independent experiments. *P* values are determined by one-way ANOVA followed by Bonferroni post-test, n.s.: not significant. Source data are provided as a Source Data file.

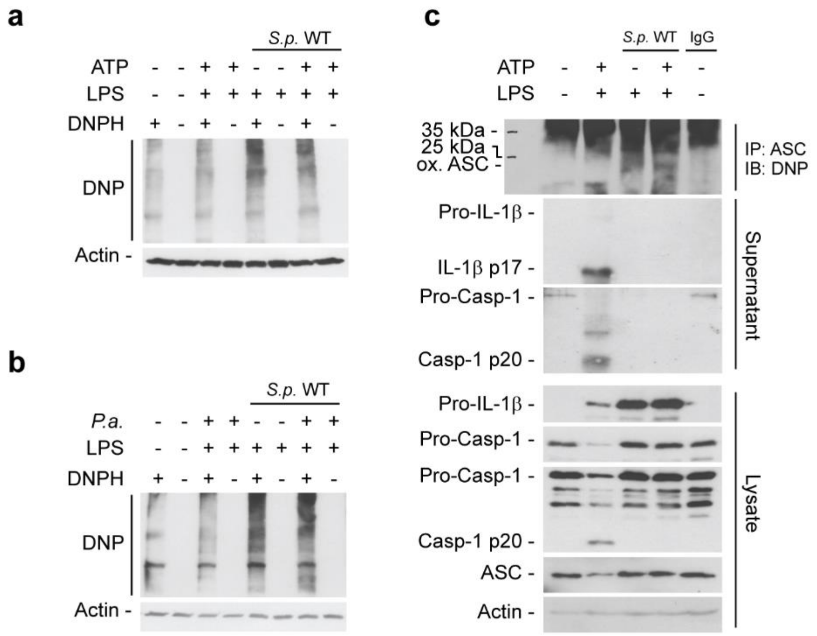

Supplementary Figure 10 *S. pneumoniae*-derived H_2O_2 induces oxidation of inflammasome components. a, **b** Immunoblot (IB) analysis of dinitrophenyl (DNP)-labelled carbonylated proteins in LPS-primed BMDMs stimulated with **a** ATP (5 mM, 30 min) or **b** *P.a.* (MOI 20, 60 min) pre-infected (or not) with *S.p.* WT (D39, MOI 50, 30 min). **c** LPS-primed BMDMs infected with *S.p.* WT (D39, MOI 50, 30 min) and then stimulated with ATP (5 mM, 30 min). IB analysed for ASC carbonylation with anti-DNP in ASC immunoprecipitates (IP). The data are representative of two independent experiments. Source data are provided as a Source Data file.
