# Supplementary material for: Hydrogen peroxide release by bacteria suppresses inflammasome-dependent innate immunity
Source: Nat Commun. 2019 Aug 2;10:3493. doi: 10.1038/s41467-019-11169-x (PMC6677825; doi:10.1038/s41467-019-11169-x)
Supplement: Supplementary file 3 — Reporting Summary [file 41467_2019_11169_MOESM3_ESM.pdf]

nature research

Corresponding author(s): Nelson Gekara

☐ Initial submission ☐ Revised version ☒ Final submission

Life Sciences Reporting Summary

Nature Research wishes to improve the reproducibility of the work that we publish. This form is intended for publication with all accepted life science papers and provides structure for consistency and transparency in reporting. Every life science submission will use this form; some list items might not apply to an individual manuscript, but all fields must be completed for clarity.

For further information on the points included in this form, see Reporting Life Sciences Research. For further information on Nature Research policies, including our data availability policy, see Authors & Referees and the Editorial Policy Checklist.

Experimental design

1. Sample size

Describe how sample size was determined.	For in vivo experiments each mouse represents one data point. A sample size of n=5 means that the data shown represent the mean value of 5 mice per experimental condition. In in vitro experiments ,each data point is a biological replicate. The number of data points was used to calculate the P value for a given experiment. Statistical methods are reported in the "Methods" section and indicated in each figure legend.

2. Data exclusions

Describe any data exclusions.	No exclusion criteria was used.

3. Replication

Describe whether the experimental findings were reliably reproduced.	Yes, on numerous independent experiments (at least two) and over an extended period of time, using several distinct approaches (as detailed in the manuscript).

4. Randomization

Describe how samples/organisms/participants were allocated into experimental groups.	Mice were sex and age matched.

5. Blinding

Describe whether the investigators were blinded to group allocation during data collection and/or analysis.	Not blinded

Note: all studies involving animals and/or human research participants must disclose whether blinding and randomization were used.

6. Statistical parameters

For all figures and tables that use statistical methods, confirm that the following items are present in relevant figure legends (or in the Methods section if additional space is needed).

n/a	Confirmed	
☐	☒	The exact sample size (*n*) for each experimental group/condition, given as a discrete number and unit of measurement (animals, litters, cultures, etc.)
☐	☒	A description of how samples were collected, noting whether measurements were taken from distinct samples or whether the same sample was measured repeatedly
☐	☒	A statement indicating how many times each experiment was replicated
☐	☒	The statistical test(s) used and whether they are one- or two-sided (note: only common tests should be described solely by name; more complex techniques should be described in the Methods section)
☐	☒	A description of any assumptions or corrections, such as an adjustment for multiple comparisons
☐	☒	The test results (e.g. *P* values) given as exact values whenever possible and with confidence intervals noted
☐	☒	A clear description of statistics including central tendency (e.g. median, mean) and variation (e.g. standard deviation, interquartile range)
☐	☒	Clearly defined error bars

See the web collection on statistics for biologists for further resources and guidance.

June 2017

Software

Policy information about availability of computer code

7. Software

Describe the software used to analyze the data in this study.	The data were plotted and statistically analysed using Graphpad prism software (https://www.graphpad.com/scientific-software/prism)

For manuscripts utilizing custom algorithms or software that are central to the paper but not yet described in the published literature, software must be made available to editors and reviewers upon request. We strongly encourage code deposition in a community repository (e.g. GitHub). *Nature Methods* guidance for providing algorithms and software for publication provides further information on this topic.

Materials and reagents

Policy information about availability of materials

8. Materials availability

Indicate whether there are restrictions on availability of unique materials or if these materials are only available for distribution by a for-profit company.	The ASC-/- mice, anti-mouse ASC and anti-mouse Caspase-1 antibodies were provided by Genentech, San Francisco USA (www.gene.com/scientists/mta)

9. Antibodies

Describe the antibodies used and how they were validated for use in the system under study (i.e. assay and species).	The anti-mouse ASC and anti-Caspase 1 were validated by comparing wild type cells and ASC and Caspase deficient cells.

10. Eukaryotic cell lines

a. State the source of each eukaryotic cell line used.	No cell lines were used for experimentation. Each experiment was conducted using macrophages freshly differentiated from mouse bone marrow progenitors or isolated from healthy human blood donors.
b. Describe the method of cell line authentication used.	Not applicable
c. Report whether the cell lines were tested for mycoplasma contamination.	The L929 cell lines, the source of macrophage colony stimulating factor (MCSF), used for differentiation of macrophages was tested and confirmed to be mycoplasma free.
d. If any of the cell lines used are listed in the database of commonly misidentified cell lines maintained by ICLAC, provide a scientific rationale for their use.	N/A

Animals and human research participants

Policy information about studies involving animals; when reporting animal research, follow the ARRIVE guidelines

11. Description of research animals

Provide details on animals and/or animal-derived materials used in the study.	Experiments were conducted using wild type and ASC-/- mice littermates on C57BL/6 background. Both male and female mice, 8-20 week of age were used. BMDMs were generated by differentiating mouse bone marrow progenitors.

Policy information about studies involving human research participants

12. Description of human research participants

Describe the covariate-relevant population characteristics of the human research participants.	Blood from healthy volunteers was used for the isolation of human PBMCs.
